# Self-Love or Other-Love? Explicit Other-Preference but Implicit Self-Preference

**DOI:** 10.1371/journal.pone.0041789

**Published:** 2012-07-25

**Authors:** Jochen E. Gebauer, Anja S. Göritz, Wilhelm Hofmann, Constantine Sedikides

**Affiliations:** 1 Humboldt-Universität zu Berlin, Berlin, Germany; 2 Universität Freiburg, Freiburg, Germany; 3 University of Chicago, Chicago, Illinois, United States of America; 4 University of Southampton, Southampton, United Kingdom; University of Western Brittany, France

## Abstract

Do humans prefer the self even over their favorite other person? This question has pervaded philosophy and social-behavioral sciences. Psychology’s distinction between explicit and implicit preferences calls for a two-tiered solution. Our evolutionarily-based Dissociative Self-Preference Model offers two hypotheses. Other-preferences prevail at an explicit level, because they convey caring for others, which strengthens interpersonal bonds–a major evolutionary advantage. Self-preferences, however, prevail at an implicit level, because they facilitate self-serving automatic behavior, which favors the self in life-or-die situations–also a major evolutionary advantage. We examined the data of 1,519 participants, who completed an explicit measure and one of five implicit measures of preferences for self versus favorite other. The results were consistent with the Dissociative Self-Preference Model. Explicitly, participants preferred their favorite other over the self. Implicitly, however, they preferred the self over their favorite other (be it their child, romantic partner, or best friend). Results are discussed in relation to evolutionary theorizing on self-deception.

## Introduction

Is the self the most cherished entity that humans possess, preferred even over their most valued other person (e.g., child, romantic partner, best friend)? Alternatively, as cultural animals [1], do humans prefer their favorite other over the self? This issue has pervaded philosophical thinking and the social-behavioral sciences ever since Aristotle [2] coined the terms ‘self-love’ and ‘other-love.’ Polarizing views on human nature as “bad” (manifested by a relative preference for self) versus “good” (manifested by a relative preference for others), this issue also lies at the heart of the Hume-Rousseau debate in Western philosophy [3], the Xunzi-Mencius debate in Confusion philosophy [4], and the Machiavelli-Botero quarrel in politics [5]. The issue is relevant to economists’ ongoing controversy on whether human decision-making is self-oriented or other-oriented [6], and it informs evolutionary discourse on individual-selection versus group-selection processes [7]. Finally, in psychology, the issue is at the heart of the Batson-Cialdini debate on the existence of true altruism [8] and the differing Baumeister-Durkheim perspectives on suicide as altruistic self-sacrifice [9].

Recent psychological advances call for a two-tiered solution to this age-old dilemma. Preferences, like self-oriented versus other-oriented ones, can be held at an explicit level; as such, they are conscious, reflective, and reasoning-based [10], [11]. Concurrently, preferences can be held at an implicit level; as such, they are automatic, spontaneous, and impulsive [10], [11]. These psychological advances illustrate the need to ask two, instead of one, questions regarding the dynamic of self-other preferences. First, are preferences for self more or less prevalent than preferences for favorite other at an *explicit* level? Second, are preferences for self more or less prevalent than preferences for favorite other at an *implicit* level? Our answers to these two questions are guided by an evolutionary model that we outline next.

### Dissociative Self-Preference Model

To address the above two pivotal questions, we propose the Dissociative Self-Preference Model. Its evolutionary backbone generates divergent hypotheses for explicit and implicit preferences. *Explicitly*, preferences for favorite other should prevail over preferences for self. Explicit other-preferences are evolutionarily adaptive, because they convey caring for others [12], which fosters interpersonal bonds [13]. Interpersonal bonds, in turn, boost evolutionary fitness [14]. *Implicitly*, however, preferences for self should prevail over preferences for favorite other. Implicit self-preferences are also evolutionarily adaptive, because they facilitate self-favoring automatic behavior in live-or-die situations [15] as well as subtle and automatic self-favoritism in everyday situations [16]. Such action maximizes self-protection [17], which, in turn, boosts evolutionary fitness [14].

We report a study in which we examine the two hypotheses derived from the Dissociative Self-Preference Model. Preferences for favorite other will predominate over preferences for self at the *explicit* level. However, preferences for self will predominate over preferences for favorite other at the *implicit* level. We test these hypotheses on a large and divergent sample, using five established implicit measures for generalizability purposes.

## Materials and Methods

### Ethics Statement

The study was run as part of a research grant awarded by the German Research Foundation (DFG; www.dfg.de) to the second author (grant identifier: GO 1107/4-1). The DFG's board of ethics passed the research proposal that underlies the present study. DFG-funded projects do not require additional approval by other ethics committees (e.g., from universities). Irrespective, we informed participants at the outset that the study concerns the psychological representation of their favorite person and that they therefore will be asked to complete corresponding self-report measures and sorting tasks. Participants were also informed that participation is anonymous, consented to participate in this study by clicking on a button, and were shown a debriefing screen at the conclusion of the study session.

### Sample and Procedure

We used a diverse internet sample in terms of age, occupation, and family status comprising 1,519 German volunteers (69% women; *M*_age_  = 37.8 years, *SD*_age_  = 13.3; this description does not include participants filtered by standard algorithms for the computation of implicit scores, such as the IAT's d-score, see Table 1). At the beginning of the study, participants provided the full name of “the person for whom you have the most positive feelings–the person who is most valuable and likable to you” (*favorite other*). Following that, we wondered whether participants had chosen themselves as their favorite other. 42 participants (3%) had done so, and we excluded their data from our analyses. Next, participants reported their own name. Subsequently, participants completed one of five implicit self-other preference measures and one explicit self-other preference measure. For each participant, we determined randomly which implicit measure (s)he had to complete. Also, for each participant, we determined randomly whether (s)he received the implicit or the explicit measure first.

**Table 1:** Key characteristics of the five implicit measures.

		IAT Sample	SBIAT Sample	GNAT Sample	APP Sample	NLT Sample
**Critical Trials**		72 self + pos & fop + neg	72 self + pos & fop + neg	36 self + pos	7 self + pos	26 letters rated
		72 self + neg & fop + pos	72 self + neg & fop + pos	36 self + neg	7 self + neg	self-initials
				36 fop + pos	7 self + neutral	fop-initials
				36 fop + neg	7 fop + pos	
					7 fop + neg	
					7 fop + neutral	
**Category-Order Randomly Varied Between Participants?**		yes	yes	yes	yes	–
**Fixation Cue**						
	**Presented?**	no	yes	no	yes	no
	**Presentation time**	–	200 ms	–	200 ms	–
	**Cue-prime interval**	–	0 ms	–	0 ms	–
**Prime**						
	**Presentation time**	–	–	–	200 ms	–
	**Prime-target interval**	–	–	–	100 ms	–
**Target**						
	**Presentation time**	until key pressed	until key pressed	700 ms oruntil key pressed	until key pressed	–
**Response-Feedback**						
	**Type**	only negative	only negative	positive and negative	no feedback	–
	**Presentation time**	until corrected	until corrected	700 ms	–	–
**Inter-Item-Interval**		700 ms	700 ms	700 ms	700 ms	–
**Scoring Algorithm**		[24]	[24]	[25]	[22]	[27]
**Implicit-Explicit Correlation**		.29***	.16***	.21***	.16*	.20***

*Note.* fop  =  favorite other person, pos  =  positive, neg  =  negative; ***p  = .001, *p  = .05.

### Explicit Measure

Participants responded to the following eight items on rating scales ranging from 1 =  “[*own full name*]” to 11 =  “[*favorite other's full name*]”: “For whom do you have…” (1) “…more positive feelings?”, (2) “…more negative feelings?” (reverse-scored), and “Who is more…” (3) “… worthy to you?”, (4) “…pleasant to you?”, (5) “…likable to you?”, (6) “…worthless to you?” (reverse-scored), (7) “…unpleasant to you?” (reverse-scored), and (8) “…unlikable to you” (reverse-scored) (α  = .89).

### Implicit Measures

Different implicit measures may assess somewhat distinct facets of self-preference [18]. To overcome this potential limitation, we administered five established implicit measures: Implicit Association Test (IAT; [19]), Single Block IAT (SBIAT; [20]), Go-Nogo Association Task (GNAT; [21]), Affective Priming Paradigm (APP; [22]), and Name-Letter-Task (NLT; [23]).

Attitude-categories of the category-based measures (IAT, SBIAT, GNAT) were “[*own full name*]” and “[*favorite person’s full name*].” Attitude-stimuli of the stimuli-based measures (IAT, SBIAT, GNAT, APP) were idiosyncratically generated by each participant. Specifically, participants were instructed to provide “three words that clearly and spontaneously signify [*favorite person’s full name*], and three different words that clearly and spontaneously signify yourself.” The instructions set two additional constraints. First, the chosen words for self and favorite other had to be equivalent (e.g., initials for self *and* for favorite other). Second, each word had to signify distinctively one attitude-category and not the other (e.g., only applicable to self, not to favorite other). Instructions also included examples suggesting the use of first names, last names, nicknames, or initials.

Valence-categories of the category-based measures (IAT, SBIAT, GNAT) were “Positive” and “Negative.” Valence-stimuli of the stimuli-based measures (IAT, SBIAT, GNAT, APP) were “worthy,” “pleasant,” and “likable” for the “Positive” category and “worthless,” “unpleasant,” and “unlikable” for the “Negative” category (the APP additionally included three neutral distracter-stimuli). These valence-stimuli were chosen to match the items of the explicit measure.

Table 1 provides a detailed description of the key characteristics of the implicit measures (e.g., number of trials, sequence of stimuli). In the IAT, participants are instructed to sort–as fast as possible–representative items to four categories. Two of these categories are the rivalry attitude-objects (here: self vs. favorite other) and the other two are opposing valence categories (here: positive vs. negative). In half of the critical trials, participants had to press the same key for sorting correctly the “self” items as well as the “positive” items, and another key for sorting correctly the “favorite other” items as well as the “negative” items. In the other half of the critical trials participants had to press the same key for sorting correctly the “self” items as well as the “negative” items, and another key for sorting correctly the “favorite other” items as well as the “positive” items. Comparison of the reaction times between the two halves of the critical trials provides an implicit index of self-other preference. Specifically, the degree to which a person is faster at trials where “self” and “positive” (and “favorite other” and “negative”) are equated, compared to trials where “favorite other” and “positive” (and “self” and “negative”) are equated, is an indicator of self-preference over other-preference [24].

In the IAT, the two halves of critical trials are administered in separate blocks, one after the other. The SBIAT is a variant of the IAT that administers all its critical trials randomly within a single block. This can address several alternative explanations of IAT effects [20]. The GNAT also builds on the IAT, but each trial has a very narrow time window in which participants can complete the sorting task. Correspondingly, the GNAT does not capitalize on response latencies to derive preferences, but on the number of errors participants make at critical trials. Specifically, the degree to which a person makes less errors at trials where “self” and “positive” (or “favorite other” and “negative”) are equated, compared to trials where “favorite other” and “positive” (and “self” and “negative”) are equated, is an indicator of self-preference over other-preference [25].

In the APP, participants are instructed to indicate–as fast as possible–whether valence items are positive or negative. Shortly before a valence item is presented, however, an attitude item (here: from the categories “self” or “favorite other”) is presented. Presentations of valence items and attitude items are randomized, so that four types of trials emerge: self-positive trials, self-negative trials, favorite other-positive trials, and favorite other-negative trials. Comparison of the reaction times between self-positive and favorite other-negative trials with favorite other-positive and self-negative trials constitutes an implicit index of self-other preference. Specifically, the degree to which a person is faster at self-positive and favorite other-negative trials, compared to favorite other-positive and self-negative trials, is an indicator of self-preference over other-preference [22]. APP is the only implicit measure in our study that does not use attitude-categories, while nonetheless using (idiosyncratically generated) attitude-items. As such, APP is sensitive to the degree to which participants have chosen attitude-items that reflect valence per se, independently of the attitude-items' link to self/favorite other. For example, the attitude-items “anxious” or “intelligent” carry valence per se (in contrast to attitude-items such as [first name] and [initials], which were provided as examples in the instructions). Thus, within the standard algorithm to compute APP scores, we embedded the exclusion of participants who–contrary to instructions–chose attitude-items that reflected valenced traits of self or favorite other (e.g., “intelligent,” “anxious”). Note that such exclusion was unnecessary in the case of those implicit measures that utilize attitude-categories (IAT, SBIAT, GNAT), because these measures are much more robust against such confounds [26].

In the NLT, participants indicate, for all letters of the alphabet, how positive or negative they perceive them to be. The degree to which participants rate their initials more positive than the initials of their favorite other is an indicator of self-preference over other-preference [27]. In order to calculate preference indices for the implicit measures, we strictly followed past research (Table 1).

## Results

To begin with, we examined the correlations between the explicit preference measure and each of the five implicit preference measures. Explicit-implicit correlations were consistently positive and significant, while also being consistently low,.16≤ *r* ≤.29, all *p*s <.05 (Table 1). This pattern buttresses the suitability of our measures, while also buttressing the importance of examining self-other preferences at the explicit as well as at the implicit level.

**Figure 1:**
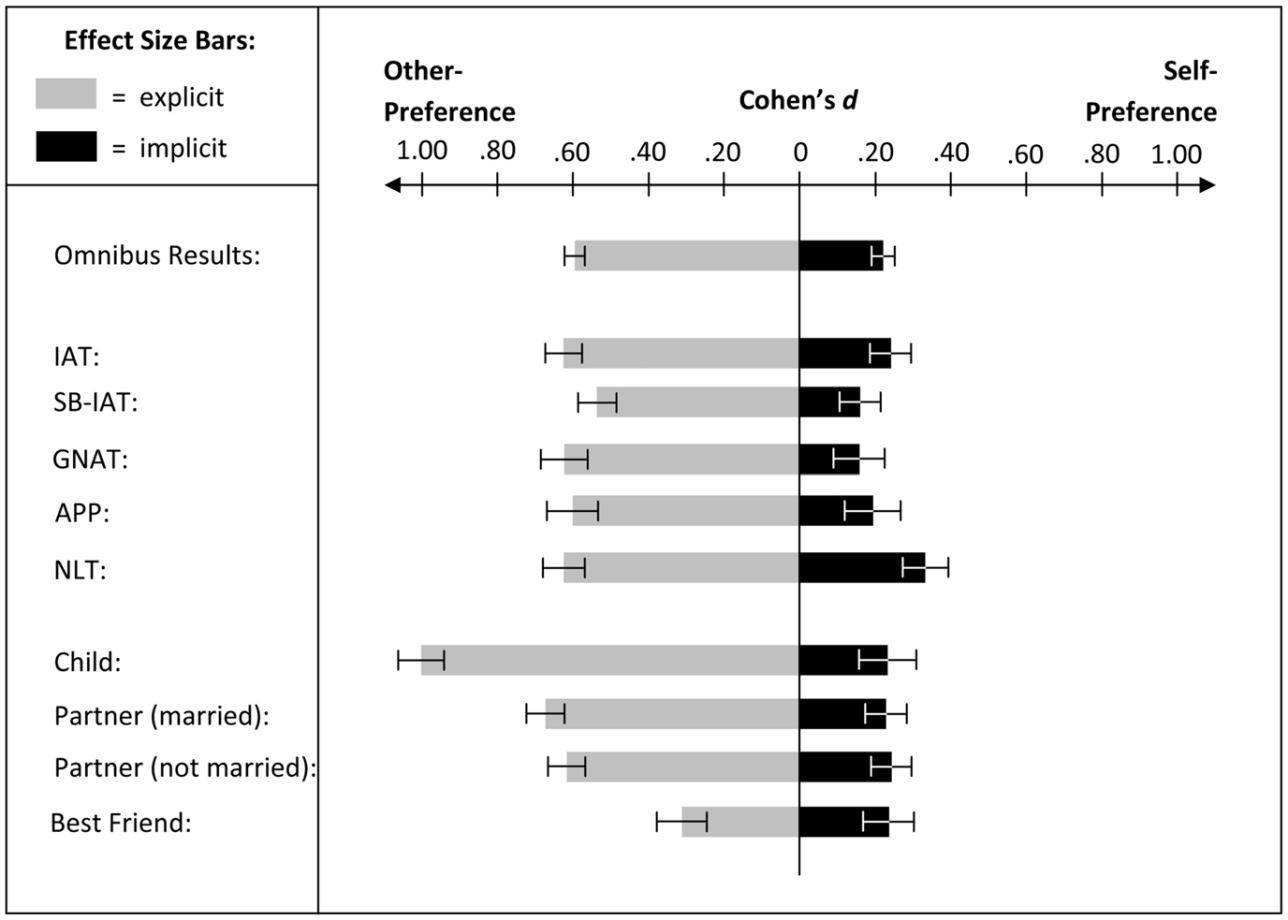
Effect sizes and standard errors of explicit and implicit preferences. All effects significant at *p*<.05.

Next, we tested the two hypotheses derived from the Dissociative Self-Preference Model. Results were fully in line with these hypotheses (Figure 1). Explicitly participants preferred their favorite other over self, as indicated by a one-sample *t*-test with the explicit measure's scale-midpoint (i.e., “6”) as comparison value, *t*(1, 1,464)  =  -23.70, *p*<.001 (*M*  = 5.09, *SE*  = .04). Implicitly, however, participants preferred self over their favorite other, as indicated by one-sample *t*-tests with the implicit measures' scale-midpoints (i.e., “0”) as comparison values, *t*(1, 380)  = 4.95, *p*  = .001 (IAT; *M*  = .25, *SE*  = .05), *t*(1, 374)  = 3.04, *p*  = .003 (SBIAT; *M*  = .16, *SE*  = .05), *t*(1, 233)  = 2.29, *p*  = .02 (GNAT; *M*  = .15, *SE*  = .07), *t*(1, 185)  = 2.59, *p*  = .01 (APP; *M*  = .19, *SE*  = .07), *t*(1, 288)  = 5.78, *p*  = .001 (NLT; *M*  = .35, *SE*  = .06).

Further, we wondered whether it is justified to treat the five implicit measures interchangeably in our subsequent analyses. Therefore, we conducted an ANOVA with type of implicit measure as the Independent Variable (IV) and standardized implicit self-preference as the Dependent Variable (DM) (i.e., implicit self-preference/SD of the corresponding implicit measure). Despite sufficiently large samples to detect reliably even small effects, this analysis revealed that the standardized effects did not differ significantly across implicit measures, *F*(4, 1,460)  = 2.03, *p*  = .09, justifying their aggregation. In line with our results for each implicit measure, the aggregate implicit index revealed a clear preference for self over favorite other as indicated by a one-sample *t*-test with the implicit index's midpoint (i.e., “0”) as comparison value, *t*(1, 1,464)  = 8.48, *p*<.001 (*M*  = .22, *SE*  = .03).

Capitalizing on the aggregate across all implicit measures, a paired-sample *t*-test revealed a significant difference between standardized explicit preferences for favorite other (*M*  =  -.62, *SD*  = 1.00) and standardized implicit preferences for self (*M*  = .22, *SD*  = 1.00), *t*(1, 1,464)  = 25.53, *p*<.001. This result buttresses the substantial difference between explicit other-preferences and implicit self-preferences. Additionally, comparison of absolute means and effect sizes (see Figure 1) shows that explicit preferences are larger than implicit preferences. This difference may date back to a weaker implicit self-preference compared to an explicit other-preference. However, this difference may (at least in part) date back to the differing nature of the implicit and explicit tasks. Evidence for the latter comes from prior research that also suggested stronger effect sizes for explicit compared to implicit preferences towards a variety of topics (e.g., flowers-insects, democrats-republicans; [28]).

Further, capitalizing on the aggregate across all implicit measures, we conducted an ANOVA with the three major types of favorite other as the IV (i.e., spouse/partner: *N*  = 751; 51%, best friend: *N*  = 245; 17%, child: *N*  = 195; 13%) and standardized implicit self-preference as the DV. Despite sufficiently large samples to detect reliably even small effects, this analysis revealed that the standardized effects did not differ significantly across type of favorite other, *F*(2, 1,151)  = .12, *p*  = .88. In contrast, an ANOVA with the three major types of favorite other as the IV and explicit other-preference as the DV was significant, *F*(2, 1,151)  = 22.05, *p*  = .001. Post-hoc analyses revealed significant differences between all three types of favorite other at *p*s <.003 (Scheffé-tests). Specifically, other-preferences were strongest for child as favorite other (*M*  = 7.42, *SD*  = 1.37), weaker for spouse/partner as favorite other (*M*  = 6.88, *SD*  = 1.31), and weakest for best friend as favorite other (*M*  = 6.52, *SD*  = 1.63) (Table 1).

People share more genes with their child than with their partner/spouse or best friend. Thus, from an evolutionary standpoint, it is conceivable that implicit preferences for self should be diminished when favorite other is one's child, rather than one's spouse/partner or best friend. Such pattern may be expected because it may maximize the survival-chances of one's genes. However, implicit self-preferences did not vary as a function of type of favorite other and a closer examination of the prehistoric parent-child relationship may provide an evolutionary explanation for our results. Specifically, in prehistoric humans both, the fertility rate as well as the child death rate were high [29]. Thus, it may well have been advantages to decisively side for self rather than for any one particular child in order to maximize survival chances of one's own genes.

### Sex and Age Differences

We tested for sex differences in explicit and implicit preferences, using a MANOVA with sex as the IV and explicit and implicit preferences as simultaneous DVs. Despite sufficiently large samples to reliably detect even small differences, we found no sex differences for explicit preferences, *F*(1, 1,458)  = 1.28, *p*  = .26, as well as for implicit preferences, *F*(1, 1,458)  = 1.08, *p*  = .30.

We also tested whether explicit and implicit preferences varied as a function of participant age. Despite sufficiently large samples to reliably detect even small effects, age was uncorrelated with explicit preferences, *r*(1,456)  =  -.03, *p*  = .23, as well as with implicit preferences, *r*(1,456)  = .01, *p*  = .66.

### On the Nature of Other-Preferences

Do explicit other-preferences reflect genuine beliefs or conscious self-presentational efforts to deceive others? Additional analyses suggest the former. First, internet studies minimize self-presentation [30]. Second, many of our current participants (*N*_1_ = 520, *N*_2_ = 793) completed two prior studies, each including a different, well-validated measure of self-presentation (datasets were linked via anonymous key-strings). Comparison of correlation coefficients using Fisher z transformation revealed that the influence of self-presentation on implicit and explicit preferences did not differ significantly with regard to the first index, z  = .74, *p * = .46 (Social Desirability Scale–17 [31]) as well as the second index, z  = .85, *p*  = .40 (Impression Management Scale [32]). Third, the influence of self-presentation on implicit and explicit preferences was generally low, -.08< *r*s < -.02. Finally, the influence of self-presentation on implicit and explicit preferences was significantly lower than the influence of self-presentation on the golden standard measure of explicit self-esteem (Rosenberg Self-Esteem Scale [33]), which we had additionally administered at the end of this study, zs  = 5.75, *p*s <.001 (comparison of correlation coefficients again utilized Fisher z transformation). Together, conscious self-presentational efforts to deceive others are unlikely to account substantially for other-preferences (Figure 1).

## Discussion

Explicitly, humans prefer their favorite other over the self. Implicitly, however, they prefer the self over their favorite other–be it their spouse/partner, best friend, or child. We consistently documented these results across five subsamples using five divergent implicit measures. The uncovered dissociative preferences may reflect ancestral traces and confer, in distinct ways, evolutionary advantages to the individual: Explicit preferences establish and cement interpersonal ties [12], whereas implicit preferences ensure automatic self-favoritism that can lead to self-preserving spontaneous behavior [15].

Von Hippel and Trivers [13] recently offered an evolutionary account of self-deception. Among other instances of self-deception, these authors suggested that “the dissociation between implicit and explicit attitudes lends itself to self-deception by enabling people to express socially desirable attitudes while nevertheless acting upon relatively inaccessible socially undesirable attitudes when they can maintain plausible deniability” (p. 7). The Dissociative Self-Preference Model is consistent with the von Hippel and Trivers account. Whereas their reasoning concerns more specific attitudes, our reasoning concerns global preferences of self over others–preferences or attitudes that are highly likely to be met with stern disapproval [34], [35]. Regardless, the current research showcases first empirical support for the Dissociative Self-Preference Model and–by extension–for von Hippel and Trivers’s evolutionary theory of self-deception. Attesting to their generalizability and robustness, the results were obtained across a large and diverse sample and across multiple implicit measures.

The present hypotheses tested *distal* evolution-based predictions–namely, the general human tendency to hold explicit other-preferences, but implicit self-preferences. Future research could examine the proximate mechanisms underlying the reported findings. For example, humans seek self-awareness in positive situations, but avoid self-awareness in negative situations [9]. This preferential paring of self with positively-valenced situations may be one such proximate mechanism. In this regard, what role may higher familiarity of self versus favorite other play in our results? After all, IAT effects need not date back to differences in category valence, but may date back to differences in category familiarity (and associated category salience [36]). However, most of our other implicit measures are robust against such familiarity/salience effects [20]. Indeed, the conceptual replication of our results across five divergent implicit measures safeguards against many (unknown) alternative explanations caused by method artifacts of any one implicit measure. We are not aware of any other study that used a similarly comprehensive approach in the involvement of implicit measures.

In conclusion, the findings are pertinent to a long-standing controversy in philosophy and the social-behavioral sciences: the dilemma of self-love versus other-love. Offering a middle-ground solution, the findings favor the arguments by Rousseau, Mencius, and Botero at the explicit level, while favoring the arguments of Hume, Xunzi, and Machiavelli at the implicit level. In Aristotle’s [2] terms, humans deep down love the self more than their favorite others; however, as cultural animals [1], they can come to believe that they love their favorite others more than they love the self.
